# Supplementary material for: Are carbon emissions trading and green financial instruments synergistic? -Comprehensive quantitative research based on content analysis
Source: PLoS One. 2024 Mar 7;19(3):e0298601. doi: 10.1371/journal.pone.0298601 (PMC10919601; doi:10.1371/journal.pone.0298601)
Supplement: S1 Data — (ZIP) [file pone.0298601.s001.zip › Data/地级市Z_F工作报告.zip!/╔╜╢1⁄2/═■║ú╩╨/└┤╫╘╣1⁄2╓┌║┼í╛┬φ┐╦╩2╛▌═°í┐_2011.docx]

各位代表：

现在，我代表威海市人民政府向大会做工作报告，请予审议，并请各位政协委员和其他列席会议的同志提出意见。

一、“十一五”时期政府工作的回顾

“十一五”时期是我市发展历程中极不平凡的五年。在省委、省政府和市委的坚强领导下，市政府团结和带领全市人民，坚持以邓小平理论和“三个代表”重要思想为指导，深入贯彻落实科学发展观，积极作为，科学务实，改革创新，攻坚克难，保持了经济平稳较快发展，保障了民生持续改善，维护了社会和谐稳定，较好地完成了“十一五”规划确定的主要目标任务。

――经济运行质量明显提高。初步统计，全市生产总值由2005年的1020.3亿元提高到2010年的1940亿元，地方财政收入由57.5亿元提高到118.3亿元，年均分别增长13.6%和15.5%。三次产业比重由10.7：58.2：31.1调整为7.5:56.7:35.8，税收、工商税收、“四税”占地方财政收入的比重分别提高2.2个、14.2个和6.9个百分点。经济发展的内生动力和结构性动力明显增强，在应对国际金融危机中表现出较强的抵御风险能力。

――经济发展方式更加集约。支持科技创新的机制进一步健全，企业的自主创新能力切实提高，传统产业改造和新兴产业培植取得突破，高新技术产业产值年均增长27.6%，占工业总产值的比重由24.9%提高到36.5%。万元生产总值能耗、水耗分别下降22%和20%，化学需氧量、二氧化硫排放量分别削减18%和6%，创新发展与绿色发展的理念得到深入落实。

――城乡统筹发展成效显著。城中村改造、农村住房建设和环境综合整治取得突破性进展，城乡基础设施全面完善，园区开发和小城镇建设快速推进，城市建成区面积由161.3平方公里拓展到240.8平方公里，城市化率由53%提高到58.7%，城市现代化和新型城镇化水平进一步提高。

――群众生活水平稳步提升。城镇居民人均可支配收入达到2.2万元，农民人均纯收入达到1万元，年均分别增长12.3%和10.6%。城乡居民人均储蓄存款达到3.7万元，年均增长15.1%。城镇居民人均住房建筑面积达到46.3平方米，农村居民人均住房居住面积达到38.9平方米，分别增加26.4%和24%。家庭轿车由3.3万辆增加到14.1万辆，宽带用户由14.6万户发展到43.2万户。城镇职工养老保险参保人数由45.2万人增加到63.4万人，城镇职工和居民基本医疗保险参保人数由40.9万人增加到122.6万人，新农保和新农合基本实现全覆盖，人民群众在发展中得到更多实惠。

五年来，我们坚持统筹兼顾，真抓实干，无论是抢抓机遇科学发展、和谐发展、率先发展，还是攻坚克难保增长、保民生、保稳定，都把主要精力用在推进转方式调结构上，用在提升发展质量和效益上，用在保障和改善民生上，努力使政府的各项工作更加符合科学发展的要求，更加符合人民群众的期待。

（一）强化结构调整，完善了更具竞争力的特色产业体系。紧紧围绕蓝色经济区和高端产业聚集区建设，积极推进农业提质增效、工业调整振兴、服务业跨越发展。全面加强农产品质量安全监管，推进出口农产品质量安全示范区建设，认证的无公害农产品、绿色食品、有机食品由101个增加到933个；省级以上农业产业化龙头企业由19家发展到50家，农民专业合作社由55家发展到968家；优质苹果、特种毛皮等特色产业形成规模，“威海刺参”、“威海无花果”、“荣成海带”、“文登大樱桃”、“乳山绿茶”等15个农产品注册为地理标志产品；粮食连年增收，总产稳定在100万吨以上，农产品年出口规模达到11亿美元，农业的产业化、品牌化和国际化程度进一步提高。按照新兴产业抓集群、传统产业创品牌的思路，狠抓了八大产业调整振兴，培植了新能源、新材料、新信息、新医药、海洋科技开发等战略性新兴产业，推进了地毯、家纺、海产品3个传统产业核心集散地建设，文登工艺家纺、荣成冷冻调理食品、荣成船舶制造获得山东省优质产品生产基地称号。造船业实现历史性突破，培育了三大造船聚集区和十大造船企业，整船制造能力由40万载重吨提高到400万载重吨，造船完工量由20万载重吨增加到120万载重吨，船舶销售收入和出口额分别扩大7.2倍和7.3倍。汽车整车制造能力由14万辆发展到30万辆，电动汽车产业的关键技术研发和配套能力建设取得突破，汽车及零配件产业集群销售收入由210亿元扩大到350亿元。新能源产业体系快速建立，石岛湾核电站和3个光伏发电项目开工建设，风电装机容量达到42.9万千瓦，新建成能源项目总装机容量达到175万千瓦，超过“十一五”前全市电力装机的总和。坚持以骨干企业、特色园区带动重点产业发展，规划建设了十大高端产业聚集园区，三角集团销售收入达到140亿元；威高集团销售收入突破100亿元，利税达到20亿元；拓展碳纤维产业化园区建成百吨级和千吨级生产线，碳纤维系列产品填补国内空白；IT产业园已有19个项目入驻。坚持陆海统筹，加快建设海洋优势产业六大基地，水产品产量由183万吨增加到210万吨，港口吞吐量由1821万吨提高到4866万吨，海洋产业增加值占生产总值的比重由25%提高到31%。整合资源推动服务业快速发展，举办了国际人居节、国际建筑设计大奖赛、渔具博览会、中俄滨海旅游度假论坛等展会，创建山东省服务名牌21个；培植了一批服务业龙头企业，家家悦销售收入突破100亿元；社会消费品零售总额达到659亿元，年均增长19.1%；旅游总收入达到221亿元，年均增长16%；服务业增加值达到695亿元，年均增长15.2%。

（二）狠抓项目建设，夯实了经济社会发展的基础。坚持把项目建设作为主攻方向，五年完成固定资产投资4700亿元，年均增长21%；组织实施市级重点项目786个，争取中央、省扩大内需项目240个。一批技术含量高、带动作用强的产业项目建成投产，其中投资过亿元的577个、过10亿元的140个，服务业、高新技术产业投资比重分别达到45.9%和37.5%。一批具有重要影响和支撑作用的基础设施项目快速推进，青荣城际铁路开工建设，威乳高速建成通车；威海港三、四期工程竣工，客运中心搬迁进展顺利，5万吨级航道建成使用；威海机场改扩建工程基本完工，机场规模扩大1倍；米威调水三期、胶东调水、烟威天然气长输管道、垃圾处理和污水处理设施等工程顺利实施，供电能力扩大1倍，供气能力扩大6.2倍，集中供热面积扩大2倍，污水处理能力扩大7倍，污水集中处理率由81.6%提高到89.3%。一批提升城市品位、增强公共服务能力的城建项目加快建设，启动了宝泉广场、金线顶、环翠楼、江家寨立交桥等区域改造工程；建成了国际展览中心、国际会议中心、文化艺术中心等一批公益设施和悦海公园、幸福公园等一批大型滨海公园；实施了文化路、世昌大道、青岛路、环山路、环海路等五路整治工程，拆除陈旧建筑210万平方米；新建改造城市道路90条、背街小巷150条，市区25条半截路全部打通；新增城市绿地面积2206公顷，衡量城市设施水平的主要指标均位居全省前列。

（三）强化自主创新，增强了区域核心竞争力。连续三年开展“自主创新年”活动，推进了高层次人才聚集区、产学研结合密集区、科技成果转化汇集区建设。累计投入13.7亿元科技创新资金，推动产学研合作，扶持企业创新平台建设。与11所高校建立了产学研战略联盟，成立了4个产业技术创新联盟，实施产学研合作项目300多项。帮助企业新建国家级研发平台8家、省级46家，总数分别达到11家和107家，争创了威高、光威2个国家级工程实验室，建立了好当家、新北洋、金猴等5个国家级企业技术中心和广泰空港国家级工程技术研究中心，新增研发平台的数量、层次和成果转化率都在全省领先。全市累计取得重要科技成果492项，三角集团巨型工程子午胎项目获得国家科技进步一等奖，威高集团聚烯烃材料改性应用等3个项目获得二等奖。高层次创业创新人才引进计划、十百千科技创新型人才培养工程顺利实施，五年引进4.6万名专业技术人才。通过创新资源集聚，一批拥有核心技术和知名品牌的企业迅速成长，新认定高新技术企业85家，科技型中小企业由33家增加到82家；中国名牌产品由8个增加到19个，中国驰名商标由7件增加到20件，三角、威高获得省长质量奖；销售收入过亿元的工业企业由575家增加到1050家，过10亿元的由31家增加到80家。

（四）坚持城乡统筹，推进了新型城镇化和新农村建设。五年来，我们把更多的精力和财力用于解决城乡建设管理中的现实问题，全市用于“三农”的财政支出累计达到163.1亿元，年均增长39.3%。在全省率先解决了城中村问题，用三年时间集中力量完成了市区77个城中村的整体改造，完成拆迁340万平方米，开工建设回迁房386万平方米，让10万城中村居民真正成为市民，市区范围内实现真正意义上的城市化。在改造过程中坚持让利于民，切实保障群众的实际利益和长远生计，并结合工程建设整治周边环境，理顺社区管理体制，使城中村改造真正成为安居工程、宜居工程、民心工程。在全省率先推进了农村住房建设工程，帮助农民新建住房10.8万户，改造危房1.4万户，166个村集中成片开发楼房4.4万户，三年的农房建设任务提前一年超额完成。启动了市区旧生活区综合改造工程，实施了400个村的环境综合整治，60%的镇建设了垃圾中转站。推动城市管理体制创新，集中治理了道路破损、停车秩序、户外广告、五小行业、散流物体运输、建筑垃圾、工地扬尘、机动车尾气、违章建筑等问题。加强了农村基础设施建设，完成了13座大中型、344座小型病险水库除险加固工程，新建农村户用沼气5万户，新增先进适用农业机械2万台，新改建农村公路2444.5公里，改造1500个村的自来水设施，完成了更高标准的村村通油路、通自来水工程。对598个经济薄弱村开展了帮扶活动。

（五）深化改革开放，增强了体制机制活力。实施了市县政府机构改革，率先启动了镇级机构改革，推进了事业单位分配制度改革，在探索大部门体制、理顺职责关系等方面取得实效。推行了部门预算、国库集中支付、政府采购和财政支出绩效评价制度，成立了政府投融资管理中心，促进了财源管理精细化、财政支出效益化、政府融资“借用管还”一体化。强化了社会综合治税，提高了税源监控的信息化水平。健全了企业融资服务机制，成立了金融工作办公室，推进了326个银企对接项目，通过建立续贷过桥周转机制为企业提供拆借资金82.5亿元，开展了小额贷款公司和科技支行试点工作，创办了3家创业投资公司，引进招商银行等23家金融机构，银行贷款余额增长2.4倍，金融生态环境更加优化。企业上市工作取得重大突破，威海广泰、华东数控、天润曲轴、新北洋、泓淋科技等7家公司在境内外上市，累计融资66亿元。理顺了市属重点国有企业和行政事业单位国有资产的管理体制。推进了集体林权和农村土地承包经营权流转制度改革。实施了文化管理体制、医药卫生体制、城市公交运营机制等社会领域改革。积极应对国际金融危机的冲击，不断创新招商方式，强化产业招商责任制，组织国内外招商活动100多次，累计引进外商直接投资25亿美元，实际利用内资1450亿元，其中投资过千万美元的外资项目157个、过亿元的内资项目320个，美敦力、摩根士丹利、安赛乐・米塔尔、现代、乐天、三星造船等世界500强企业落户威海，招商引资的规模和质量都有新提升。强化国际市场开拓资金等扶持政策，千方百计稳定外需，外贸进出口总额由2005年的75.5亿美元提高到139.2亿美元，出口产品结构和市场结构都得到进一步优化。深入推动企业战略合作，67家企业到境外投资发展，180家企业与国际知名企业战略重组。圆满完成对口援川、援青、援疆等帮扶任务，强化外事、侨务、对台工作的联络功能，区域对接合作更加紧密。

（六）加强区域统筹，构建了协调共进的发展格局。强化战略规划与资源整合，拉开了一体化威海建设的大框架。中心市区启动了双岛湾休闲旅游度假城、临港产业服务区建设。设立了威海工业新区，理顺了管理体制和权限，举全市之力打造新板块，引导城市向腹地拓展、项目向园区集中。工业新区两年完成基础设施投资30多亿元，引进过亿元的大项目160多个，成为外来投资的密集区和产业发展的隆起带。高区加强了科技新城开发，经区推进了临港工业园建设，发展空间都有新拓展，发展层次都有新提升，辐射带动作用都有新提高。荣成石岛管理区、文登南海新区、乳山海湾新区、环翠张村旅游度假区的开发建设都展现出新手笔，聚集功能和集约发展水平明显增强。县域经济的发展活力和综合实力不断提高，13个中心镇加快发展，区域经济格局进一步优化。

（七）重视生态文明，加强了资源环境保护。坚持把生态环境作为最大的竞争优势来保护，把宜居城市作为最靓的品牌来打造。大力推进节能减排，加强了重点用能企业和排污企业的在线监测系统建设，关停“小火电”27.1万千瓦，淘汰落后水泥产能124.3万吨，圆满完成了“十一五”节能减排任务，城市环境综合整治定量考核、污染减排和环境改善考核连续保持全省第一。高度重视资源的保护和集约利用，强化了土地、海域、岸线、沙滩、山体和城市绿地规范化、制度化管理，推进了市区河道、重点流域和饮用水水源地的综合整治，关停整合各类矿山414个。加强了林业病虫害防治，成片造林50万亩。获得“国家森林城市”、“中国人居环境奖”、“全国节水型城市”、“全国绿化模范城市”、“生态省建设先进市”等荣誉称号，荣成市建成国家生态市，文登市、乳山市分别通过验收、评估。

（八）突出文化建设，促进了各项社会事业协调发展。健全了文化产业推进机制和公共文化服务体系，建成16个文化产业示范基地，在文化旅游、广播电视、新闻出版、影视动漫等领域培育了一批重点企业，打造了梦海、神游华夏等文化演艺品牌，刘公岛被命名为“国家文化产业示范基地”。坚持市场化运作，承办了长距离铁人三项世界杯、霍比世界帆船锦标赛和十一届全运会相关项目，以品牌赛事推动体育产业发展，全民健身活动深入开展。实施了五大文化惠民工程，开展了幸福威海系列文化活动和中韩文化交流月活动，加强了镇综合文化站、农村文化大院、农家书屋建设，乳山市徐家镇创建了全国先进基层文化站。深入挖掘史志、档案等文化资源，加强了文物和非物质文化遗产保护。结合创建全国文明城市，开展了形式多样的群众性精神文明创建活动。加强了国防动员、民兵预备役和双拥共建工作，获得“全国人民防空先进城市”称号。计划生育奖励扶助政策惠及24万人，继续保持低生育水平，被评为“全国人口和计划生育综合改革示范市”。民族、宗教、地震、气象等事业也取得新成绩。

（九）充分关注民生，完善了民生保障体系。过去的五年，是我市民生政策出台最密集、人民群众得到实惠最多的时期之一。在落实国家、省各项政策的同时，我们充分考虑群众的需求、威海的实际和财政保障能力，最大限度地通过机制创新来扩大覆盖面、提高保障标准。在就业领域，建立健全了失业人员、大龄就业困难群体、大中专毕业生等就业帮扶机制，开展了国家级创业型城市创建活动，完善了鼓励自主创业的税费减免和担保贷款等政策，新增城镇就业22万人，转移农村劳动力10.1万人。在教育领域，出台了加强农村教育、素质教育、学前教育和职业教育等一系列政策措施，新建扩建学校175所、镇中心幼儿园53所，财政性教育经费年均增长27.5%，城乡全面实行了真正的免费义务教育，教育综合水平保持全省前列，被评为“全国义务教育均衡发展工作先进地区”。在社会保障领域，率先在全省实现国家试点标准的新农保全覆盖；率先将大龄城镇居民纳入养老保障范围，惠及5.6万人；率先建立城镇居民医疗保险制度，解决了43.1万人的医保问题。在医疗卫生领域，加强了标准化村卫生室和城市社区卫生服务机构建设，城镇职工医保最高支付限额由13万元提高到24万元，城镇居民医保和新农合最高支付限额分别提高到13万元和5万元。在社会救助领域，健全了困难家庭学生资助、大病救助、住房救助、临时救助等以政府为主导的社会救助体系，城乡低保对象扩大到5.5万人；在全国率先建立起城乡一体化的孤残儿童保障机制，开展了形式多样的慈善救助活动。在住房保障领域，建立了经济适用房配建和廉租房货币化补贴制度，建设经济适用房6078套、廉租住房556套，发放补贴363万元。在保障群众权益领域，强化民主法制建设，推进了普法依法治理、人民调解、仲裁、信访、法律服务、法律援助工作，强化了社会治安综合治理，加强了食品药品和安全生产监管，各类安全事故下降51%；健全了应急管理体系，有效应对甲型H1N1流感、手足口病、问题奶粉、风暴潮、浒苔、海冰等突发事件，保障了人民群众的生命财产安全。

（十）转变政府职能，优化了行政服务环境。结合深入学习实践科学发展观和创先争优活动，扎实开展了“问政于民、问需于民、问计于民”、“向市民承诺、为市民办事、请市民监督”等活动，加强了服务型政府建设。强化“企业创造财富，政府营造环境”、“院内的事企业干，院外的事政府办”的理念，推行领导分包和企业特派员制度，加强了对企业和项目的扶持。制订了扶持企业做大做强、促进中小企业发展、奖励纳税明星和新星企业等政策措施，取消调整收费项目263项，最大限度地减负让利。坚持依法行政，强化了政府信息公开，推进了相对集中部门行政许可权工作，完善了覆盖全市的电子监察网络。健全了廉政风险防范管理制度，加强了政府投资项目等专项督查。自觉接受人大的法律监督、政协的民主监督，定期向人大和政协报告工作，办理人大代表建议和政协委员提案1025件。强化目标绩效管理考核，开展了行风热线、民主评议政风行风工作，严格执行“禁酒令”等纪律规定，机关作风进一步转变。

各位代表！刚刚过去的一年，全市上下深入贯彻落实科学发展观，把保持经济平稳较快发展与转方式调结构紧密结合起来，紧紧围绕蓝色经济区和高端产业聚集区建设，强化自主创新、项目建设、产业培植和企业扶持，实施了旅游文化产业发展、保护中小企业发展、新型城镇化建设和城市精细化管理“四大行动”，有效巩固和扩大应对国际金融危机冲击成果，经济保持良好的增长势头，各项社会事业和民生保障工作也得到加强。初步统计，全市生产总值增长12.2%，地方财政收入增长15.4%，经济运行质量和效益进一步提高。固定资产投资增长17%，社会消费品零售总额增长18.5%，扩大内需的一系列措施取得积极成效。外商直接投资增长3%，实际利用内资增长12%，利用外资结构进一步优化。外贸进出口增长31.1%，对外贸易总量超过危机前水平。万元生产总值能耗降低4.18%，化学需氧量、二氧化硫排放量分别削减1.5%和1%，资源环境的管理保护机制进一步健全。城镇居民人均可支配收入增长10.5%，农民人均纯收入增长9.2%，在医药卫生、社会保障、教育、文化等领域都推进了体制机制创新，进一步改善了民生，促进了社会和谐稳定。

各位代表！过去的五年，是我市经济社会发展取得丰硕成果的五年，也是在各种复杂环境和严峻挑战中奋力前行的五年。五年的实践启示我们，要不断开创科学发展、和谐发展、率先发展的新局面，必须坚定不移地与党中央保持高度一致，坚决贯彻执行省委、省政府决策部署和市委决议，充分运用各种有利条件，从容应对各种困难和挑战，聚精会神搞建设，一心一意谋发展；必须大张旗鼓地鼓励和保护创新精神，以思想解放凝聚合力，以深化改革激发活力，以资源整合挖掘潜力，把比较优势充分发挥出来，为经济社会发展注入强大动力；必须统筹兼顾地处理经济与社会发展、城市与农村发展、人与自然和谐发展等一系列重大关系，促进各方面发展更加均衡、更加协调；必须大力弘扬求真务实、真抓实干的作风，讲实话、办实事、出实招、求实效，把对企业和群众的服务体现到狠抓落实上；必须全心全意地为人民群众谋福利，始终做到尊重人民、敬畏人民、感谢人民、服务人民、依靠人民，最大限度地调动全市人民的积极性和创造性。

各位代表！五年的发展成果来之不易，全市上下为此付出了艰辛努力。市委总揽全局，掌舵把关，谋划发展方略，凝聚各方力量；市人大依法监督、鼎力支持，市政协民主监督、参政议政，并且都积极做好分包项目和联系企业工作，为威海的发展加油助力；市纪委、法院、检察院多方支持，保驾护航；驻威部队官兵携手共建，无私奉献；社会各界和全市人民对政府工作也给予了大力支持。在此，我代表市人民政府，向全市人民，向各位人大代表和政协委员，向各民主党派、工商联、各人民团体和社会各界人士，向驻威部队指战员、武警官兵和公安干警，向各位老领导、老同志，向所有关心支持威海发展的海内外朋友，致以崇高的敬意和衷心的感谢！

各位代表！在总结成绩的同时，我们也清醒地看到，我市经济社会发展中还存在一些矛盾和问题，主要表现在：经济运行质量和效益有待进一步提高，结构性矛盾还比较突出，现代服务业发展相对滞后，自主创新能力有待增强，发展方式需要加快转变；环境保护的压力不断加大，部分资源无序开发的问题有待规范，生态文明建设需要进一步加强；城乡居民收入差距仍然较大，农村生产生活条件和公共服务水平相对落后，城市管理体制还需理顺，统筹城乡发展的力度需要进一步加大；在就业、教育、住房、医疗卫生、社会保障等关系群众切身利益的领域还存在不少难点、焦点问题，改善社会民生任务艰巨；政府部门的工作作风、行政效能与发展要求还有一定差距，反腐倡廉力度需要进一步加大，政府自身建设仍需加强。对此，我们一定高度重视，采取有针对性的措施，在今年和“十二五”的工作中认真加以解决。

二、“十二五”时期的指导思想和目标任务

各位代表！“十二五”时期是全面建设小康社会的关键时期，也是深化改革开放、加快转变经济发展方式的攻坚时期，经济发展长期向好的趋势没有改变，但面临的矛盾和问题也不少，国际金融危机影响并没有结束甚至可能出现反复，我们既面临难得的历史机遇，也面对诸多可以预见和难以预见的风险挑战。为更好地指导“十二五”时期各项工作，按照国家、省的部署和市委的要求，市政府编制了《威海市国民经济和社会发展第十二个五年规划纲要（草案）》。《纲要（草案）》及《说明》已印发大会，请一并予以审议。

综合考虑各方面因素，确定“十二五”时期我市经济社会发展的指导思想是：高举中国特色社会主义伟大旗帜，以邓小平理论和“三个代表”重要思想为指导，深入贯彻落实科学发展观，以科学发展为主题，以加快转变经济发展方式为主线，以提高质量效益为中心，以建设蓝色经济区和高端产业聚集区为抓手，着力加强自主创新，着力促进产业振兴，着力强化内需驱动，着力深化改革开放，着力统筹城乡发展，着力建设生态文明，着力改善社会民生，力争率先全面建成小康社会，为建设创新开放宜居幸福的现代化新威海奠定坚实基础。

主要目标是：生产总值年均增长11%，三次产业比重调整为4.5：52.5：43；地方财政收入年均增长13%；城镇居民人均可支配收入和农民人均纯收入年均分别增长10%；万元生产总值能耗降低17%，化学需氧量、氨氮排放量分别削减12%左右，二氧化硫、氮氧化物排放量分别削减16%左右；固定资产投资年均增长15%，社会消费品零售总额年均增长17%，外贸进出口总额年均增长10%；累计利用外商直接投资30亿美元以上，实际利用内资年均增长15%；城市化率达到65%。

“十二五”时期的政府工作将突出以下几个重点：

第一，坚持以提高质量效益为中心，力争在推进科学发展上有新作为。正确处理速度与质量效益的关系，进一步形成质效优先的经济发展导向，健全完善以提高效益为核心的产业调整、项目建设、企业扶持等政策和目标体系，努力在提高质量效益的前提下保持经济平稳较快发展，把经济发展的成果体现到财税增收、企业增效、居民收入稳步增长上，真正体现科学发展的主题。

第二，坚持以转变发展方式为主线，力争在产业结构升级上有新跨越。围绕建设蓝色经济区和高端产业聚集区，抓好海洋优势产业六大基地、十大高端产业聚集园区建设，加快培育能够发挥比较优势、体现威海特色、具有竞争力的新型产业体系。战略性新兴产业，重点培育新能源、新材料、新信息、生物医药、新能源汽车、节能环保、海洋科技开发等七大产业；区域特色产业，重点发展现代渔业、船舶修造、港口物流、海产品精深加工等四大现代海洋产业；传统优势产业，重点打造地毯、家纺、海产品、渔具、医疗器械等五大核心集散地；现代服务业，重点发展文化、旅游休闲、软件及服务外包、商务会展、金融等五大产业。

第三，坚持以统筹城乡发展为重点，力争在区域发展格局上有新突破。扎实推进城市现代化和新型城镇化，构筑“一核一带三组团”的城镇空间格局，打响“蓝色休闲之都，世界宜居城市”的品牌。中心城市加快推进“两城两区”建设，实施好“一轴三带三中心”等重点工程。荣成市抓好“一城两带三片区”建设，文登市抓好“两大板块三大功能区”建设，乳山市抓好“两城区两新区”建设。搞好小城镇建设，打造沿海千公里城镇带。加快推进新农村建设，切实改善农村生产生活条件，有序推动本地农村人口转移，促进城乡一体化发展。

第四，坚持以科技教育人才为支撑，力争在自主创新能力上有新提升。深入实施科教兴威和人才强市战略，充分发挥科技第一生产力和人才第一资源作用，提高教育现代化水平，深入推进高层次人才聚集区、产学研结合密集区、科技成果转化汇集区建设，推动经济社会发展更多依靠科技进步、劳动者素质提高和管理创新，加快建设创新型城市。

第五，坚持以生态文明建设为基础，力争在人居环境改善上有新进步。牢固树立绿色发展理念，加大节能减排力度，努力降低能源消耗强度和污染物排放强度。健全环境保护和治理机制，实施最严格的资源管理保护制度，强化海洋资源和生态环境保护，加强城乡环境综合整治，争取在全省率先建成国家生态市。

第六，坚持以深化改革开放为动力，力争在增强发展后劲上有新成效。坚持解放思想、改革创新，进一步破除观念障碍，清除体制壁垒，增创发展优势。着力推动经济体制、社会体制和行政体制改革，进一步理顺有利于科学发展、有利于保障民生、有利于促进和谐的体制机制。实行更加积极主动的开放战略，推动对外贸易转型，科学引导外资投向，提高利用外资的质量和效益，在更高层次、更广领域打造对外开放的新优势。

第七，坚持以保障改善民生为导向，力争在公共服务能力上有新提高。注重长效机制建设，着力解决事关人民群众切身利益的民生问题，构建社会化程度更高、覆盖范围更广、保障力度更大的社会保障安全网。强化社会建设，大力推进基本公共服务均等化，协调发展各项社会事业，让人民群众享受更加优质的公共服务。创新社会管理机制，推进平安威海建设，努力化解社会矛盾，促进社会公平正义，维护社会和谐稳定。

第八，坚持以重点项目建设为保障，力争在发展环境上有新改善。深入实施项目带动战略，统筹建设一批基础设施项目，重点实施青荣城际铁路、荣乌高速荣文段、内环快速路等交通项目，泊于水库等水利项目，石岛湾核电站等能源项目以及城市公交、污水垃圾处理等市政设施项目，打造适度超前、功能完善、配套协调、高效安全的支撑保障体系，为科学发展、和谐发展、率先发展创造良好的环境。

各位代表！2011年1月4日，国务院批复了《山东半岛蓝色经济区发展规划》，山东半岛蓝色经济区正式上升为国家发展战略，这是我市发展进程中的又一重大机遇。我们将深入学习胡锦涛总书记重要论述和国务院批复精神，按照省委、省政府的战略部署，把蓝色经济区建设作为转方式调结构的有效途径，作为提高改革开放水平的强大动力，作为促进区域协调发展的重要举措，发挥优势，抓住关键，高点起步，创新发展，积极探索海洋经济科学发展的新路子、海洋资源科学开发利用的新模式和海洋生态环境保护的新途径，加快培育战略性海洋新兴产业，构筑现代海洋产业体系，提升海洋经济辐射带动能力，努力把我市建设成为全国重要的海洋产业基地、对外开放平台和我国北方独具魅力的滨海休闲度假区。

三、2011年政府工作安排

各位代表！今年是中国共产党成立90周年，也是实施“十二五”规划的开局之年，做好各项工作意义重大。全年主要预期目标为：生产总值增长11%，地方财政收入增长13%，万元生产总值能耗降低3.66%，化学需氧量、氨氮、二氧化硫、氮氧化物排放量分别削减2%左右，固定资产投资增长17%，社会消费品零售总额增长17%，外商直接投资稳定增长，实际利用内资增长15%，外贸进出口总额增长10%，城镇居民人均可支配收入增长10%，农民人均纯收入增长10%。今年政府工作的主要任务是：

（一）加快推进产业结构调整。全力培植支柱产业、特色产业、新兴产业，推动蓝色经济区、高端产业聚集区建设。

提升农业现代化水平。落实好“菜篮子”负责制，稳定粮油蔬菜播种面积，保障农产品有效供给。大力发展特色高效农业，推广苹果提质扩量增效工程，扩大特色农产品规模种植，提高农产品精深加工水平。推进现代畜牧示范区建设，规划建设交易市场，搞好品种改良，提升特种毛皮等特色养殖业的集聚发展能力。推进农超对接、基地直供，完善农产品和农资新型经营服务体系。深入推进出口农产品质量安全示范区建设，新建标准化基地8万亩，新认证无公害农产品、绿色食品、有机食品50个，树立农产品质量安全最放心城市形象。推广应用先进适用农业机械，进一步提升农机化作业水平。培育壮大一批促进农村就业、带动农民增收的农业龙头企业，鼓励发展农民专业合作社，推动发展休闲观光农业，不断提高农业的产业化、规模化、组织化水平。

推进制造业转型升级。以高端高质高效为发展方向，大力培植战略性新兴产业，改造提升传统产业，培植壮大优势产业集群。新能源产业，重点抓好石岛湾核电站和光伏发电项目，发展核心装备、关键器件等相关配套产业；新材料产业，推进医用高分子材料、碳纤维复合材料、铝镁合金材料、功能玻璃、子午线轮胎等特色材料产业化基地建设；新信息产业，抓好特种打印机、集成电路、通信产品、物联网、软件开发和信息服务业；生物医药产业，建设好华夏、迪沙、达因、文登南海四大医药园区；新能源汽车产业，推进广泰、中航黑豹电动车产业园建设，加快产业化步伐。继续实施好船舶、汽车及零配件、机械装备制造、轻工纺织等八大产业调整振兴规划，推动制造业集中集约集聚发展。强化骨干企业的带动作用和中小企业的配套功能，支持行业龙头企业兼并重组，提高产业集中度、分工层次和整体竞争力。大力实施质量兴市和名牌战略，支持企业加强品牌和标准化建设，引导行业共同打造各类地理标志品牌，提高我市产品的影响力。推动工业化与信息化融合，加快电子商务、现代物流等先进适用技术向传统优势产业渗透，扎实推进五大核心集散地建设，促进优势产业由“加工基地型”向“产业聚散型”转变。

推动现代服务业跨越发展。全面落实扶持政策，按照不低于上年度生产总值万分之一的比例设立引导资金，服务业增加值比重提高2个百分点左右。扎实开展旅游文化产业发展行动，科学开发利用文化资源，积极培育新兴文化业态，扶持影视动漫、工业设计、娱乐演艺等创意产业项目。强化旅游规划的指导作用，推进旅游资源整合和产业链建设，加强整体品牌宣传和旅游市场营销，搞好特色旅游产品开发，推动华夏生态园、汤泊温泉、大乳山二期、小汤温泉、西霞口湿地公园、阳光新地等旅游项目建设，实施国际会展中心五星级酒店、荣成万国城、文登华玺等高档酒店项目，加快滨海休闲旅游度假基地建设，旅游总收入增长15%。办好国际建筑设计大奖赛、第六届国际人居节、第四届渔具博览会、中国时装设计大赛、温泉节等会展活动。大力发展生产性服务业，鼓励企业加快非核心业务剥离，推进高区、经区2个省级国际服务外包示范基地建设。加快发展生活性服务业，大力推进“便利消费进社区，便民服务进家庭”工程，积极推进宝泉广场、金线顶、银座商城、九龙城休闲购物广场、高新商贸广场、东城国际、生态汽博城、文登大润发商城等高端商贸项目，培植壮大社区和家庭服务业，进一步扩大消费需求。在园区建设中搞好服务业布局规划和设施配套，抓好服务业的招商引资，支持企业培育服务业品牌。

发展现代海洋产业。强化海洋科技开发和海洋资源保护，加快推进海洋战略性新兴产业、现代海洋渔业、现代海洋制造业和现代海洋服务业基地建设。现代海洋渔业，突出抓好品牌建设，以“威海刺参”为重点，在市场营销、品牌推广、质量监管和产业推进上狠下功夫，打响威海海珍品的品牌；拓展现代渔业发展空间，完善养殖水域滩涂规划，扩大无公害水产品生产规模，抓好水产原良种场和示范基地建设，良种覆盖率提高3个百分点；深入实施渔业资源修复，加大海洋生态牧场的培育力度；支持企业建立综合性远洋渔业基地，提高远洋渔业产业化水平和综合竞争力；加快发展海洋食品、保健品、药品和海洋新材料产业，规划建设海产品交易中心，提高海产品的深加工能力和集散能力。现代海洋制造业，突出抓好造船产业集群培植，推动重点造船企业加强高附加值船型的研发设计，提高本地整船总装比例，加大配套企业的引进力度，提高市场占有率，造船完工量增加15万载重吨。现代海洋服务业，突出抓好临港物流产业，推动港口资源整合与战略合作，统筹抓好威海港、石岛港、龙眼港、蜊江港、张家埠港、乳山口港的发展，推进威海至仁川陆海联运汽车甩挂运输项目和石岛至高雄直航项目，引导对外开放口岸完善设施功能，优化“大通关”环境，港口货物吞吐量增长12%；推进威海港物流中心、荣成冷链物流基地、文登胶东农资物流中心、环翠区临港科技园区等项目，物流业增加值增长10%；建设休闲渔业示范点和海钓、游艇等海上休闲运动基地，促进休闲渔业快速发展。海洋战略性新兴产业，突出抓好海洋科技创新，加强与威海海洋研究院等科研院所的对接，推进海洋先进实用技术开发和核心技术攻关，打造海洋科技开发和科技成果转化的重要基地。

（二）加强重点项目建设。着眼于扩大内需，完善跟踪服务和督查机制，实施383个重点调度项目，年内完成投资898亿元；集中推进100个市级重点项目，年内完成投资268.1亿元；在社会民生等领域筛选19个调控项目，年内完成投资101.5亿元。

高端制造业领域，重点安排29个项目，年内完成投资96亿元。狠抓十大高端产业聚集园区建设，推动威高集团医疗器械园区、拓展碳纤维产业化园区、三角全钢载重子午线轮胎园区、华东数控重型数控机床产业园区、IT产业园区、新船重工黄海造船整船研发及零部件配套生产园区、中航黑豹天润曲轴汽车产业园区等各大园区尽快形成规模优势。组织实施好63个高新技术和现代制造业项目，提升制造业竞争力。

基础设施领域，重点安排14个项目，年内完成投资77亿元。实施青荣城际铁路、威海港国际客运中心、烟海高速乳山段、江家寨立交桥、统一路南延、俚李线改造等交通项目，推进胶东调水配套工程、市区燃气热力工程和4个输变电工程项目，进一步提高基础保障能力。

城建工程领域，安排16项重点工程，年内完成投资153亿元。加快推进双岛湾、工业新城等“两城两区”项目，实施金线顶、国际展览中心绿轴园林、九龙湾、环翠楼等区域开发项目，继续推进世昌大道、青岛路、文化路等综合整治项目，进一步增强承载和服务功能。

社会民生领域，重点安排16个项目。加快城中村改造回迁安置房建设，新开工建设回迁房22.4万平方米，回迁安置6211户，力争年内全部完成回迁安置任务，并做好村改居工作。实施市区25个旧生活区综合改造工程。强化市区路网建设及升级改造，完成古寨东路南延、宫松岭路等47条道路的新建改造。启动市图书馆新馆、方志馆、群众艺术馆等文化设施项目建设，市文化艺术中心投入使用。强化中小学校舍安全工程建设，新建和加固校舍17万平方米，新建扩建城区学校6所，完成威海四中迁建工程，启动中小学校外实践基地建设。

（三）增强自主创新能力。继续深入开展“自主创新年”活动，实施“创新威海科技行动”。

加强科技创新平台建设。继续推进产学研合作，争取再与2所高校建立产学研战略联盟。支持企业间加强研发合作，组建产业技术创新联盟，创建省级以上试点联盟1家。积极争取创新平台列入国家计划，推进寻山集团创建国家海产贝类工程技术研究中心，新增市级以上企业技术中心和工程技术研究中心20家，其中省级4家。强化13个科技创新公共服务平台管理，建立资源集成与开放共享机制，为企业创新创业提供技术支持。

推进科技成果产业化。深入推动科技支行试点工作，培育壮大创新型企业规模，新增高新技术企业8家、科技型中小企业15家、省级以上创新型企业3家。加快建设科技企业孵化器，推进全国先进复合材料高新技术产业化基地建设。组织好国家知识产权试点市建设，实施科技攻关计划和技术创新项目400项。

强化创新人才培养和引进。全面落实人才奖励政策，组织实施62个国外引智项目，搞好留学人员创业园、高校毕业生创业孵化基地等各类载体建设，加快引进战略性新兴产业、现代服务业等领域急需的领军人才和研发团队。扎实推进“金蓝领”培训工程，培养高技能人才3000人。

（四）推进全方位开放。深入实施合作共赢的开放战略，统筹利用国际国内两个市场、两种资源，推动开放型经济转型升级。

深化战略合作，提升招商引资层次。以日韩、欧美、港台、京沪为重点，组织实施好17次境内外重点招商活动，加强与世界500强企业、上市公司、大型央企民企的合作，支持企业引进战略投资者。鼓励推动企业境外上市，有效利用私募基金，做好产业资本与金融资本结合的文章。进一步改进产业招商办法，有针对性地引进产业链各环节的重点企业和配套企业，助推优势产业集群膨胀。建立选商择资综合评价体系，引导资金投向高端制造业、现代服务业和生态高效农业领域。完善重点项目落地跟踪服务机制，优化投资发展环境。

优化出口结构，转变外贸发展方式。深入实施市场多元化战略，积极开拓新兴市场，巩固传统市场。强化自主品牌建设，加强出口优势产品的包装宣传，提高附加值和竞争力。培育壮大外贸出口主体，进一步提高高新技术产品、机电产品的出口比重。大力发展服务贸易，提高加工贸易的本地化配套比例。引导企业扩大先进技术、关键设备、大宗资源性商品进口。

强化主动对接，推进区域经济合作。积极稳妥地推动企业“走出去”，打造橡胶、木材、矿产等境外资源基地，支持企业收购优质资产、品牌、技术及网络。加强服务平台建设，推进对外工程承包，提升外派劳务层次。积极参与中日韩自贸区建设，争取更多先行先试政策支持。充分利用外事、侨务、对台资源,扩大经贸文化交流。健全与重点区域的合作机制，加强与长三角、珠三角、环渤海经济圈及资源大省的对接，继续做好对口帮扶工作。

（五）深化重要领域改革。坚持以改革创新推动工作开展，进一步理顺有利于科学发展的体制机制。

深化财税体制改革。落实国家财税体制改革各项部署，继续推进预算制度改革，健全财政支出绩效评价机制，推动财政资金向社会民生领域倾斜。发挥融资平台的作用，做好国家、省扶持项目的衔接工作。加强财源建设，完善社会综合治税网络，规范跨区企业税收管理，提高税收征管质量，促进财政收入速度、质量双提高。力争全年地方财政收入占生产总值的比重提高0.1个百分点，税收、工商税收、“四税”占地方财政收入的比重分别提高2个、2个和0.5个百分点。

深化金融体制改革。加强社会信用体系建设，大力推进银企对接，扩大续贷过桥周转机制覆盖面，规范整顿融资性担保公司。引导异地银行积极授信，扩大小额贷款公司试点，争取引进2家外地银行设立分支机构。加快推进农村信用社银行化改革，新设2家村镇银行。加强上市后备资源培育，加快吉威医疗、俚岛海洋科技等企业上市进程，推动上市公司再融资。积极稳妥地参与区域性股权交易市场发展，推进高区“新三板”试点，拓展债券、股权等多元化融资渠道。

深化价格领域改革。全面加强价格调控监管工作，建立完善重要消费品价格的监测、预警和发布制度，做好重要农副产品的储备和市场调控，推进资源环境价格改革，保持区域价格稳定。健全社会救助和保障标准与物价上涨挂钩的联动机制，适时发放临时价格补贴，保障困难群众基本生活。

深化国有企业改革。进一步完善市属国有企业国资监管体制，积极推动企业集团建立现代企业制度，加快推进18户国有集体中小企业改制。健全行政事业单位国有资产管理使用长效机制，实现国有资产保值增值。

深化事业单位改革。按照政事分开、事企分开的原则，积极推进事业单位分类改革。搞好事业单位绩效工资制度改革，进一步完善事业单位岗位管理制度、公开招聘制度和全员聘任制度。

完善中小企业促进机制。扎实开展保护中小企业发展行动，进一步完善政策扶持体系、信用担保体系和社会化服务体系，做好特派员联络中小企业工作，深入实施中小企业成长、小企业培育、技术创新、特色提升“四项计划”，新建10家市级公共技术服务平台，重点培育19处市级小企业创业基地。

（六）加快社会主义新农村建设。坚持统筹城乡一体化发展，切实加大“三农”投入，引导更多的资源向农村倾斜，进一步增加农民收入，改善农村面貌，提升农民的生活质量。

拓宽农民增收渠道。全面落实各项强农惠农政策，健全财政支农稳定增长机制，扩大农业保险实施范围，组织创建10个以上农业科技示范园区，支持农民搞好生产经营。组织实施农民职业技能培训“阳光工程”，提高农民创业创收能力。

推进农村住房建设和环境综合整治。强化财政资金补贴，稳妥推进农村新型集中居住区建设，帮助农民新建住房3.2万户，改造危房2000户，完成300个村的环境综合整治，切实改善农村环境。

加强农村基础设施建设。实施好新一轮农村电网改造升级工程，提高县域电网的供电能力和装备水平。新建小型农田水利工程154项，发展节水灌溉面积6.2万亩。新建沼气综合利用示范基地15个，增加户用沼气3000户。完善农村设施管护长效机制，建立常态化的管护队伍和服务网络，对老化失修的农村公路和自来水工程实施更新改造。继续实施“万村千乡市场工程”和“新农村现代流通服务网络工程”，加快农村社区综合服务中心建设。

完善农村发展体制机制。坚持和完善农村基本经营制度，因地制宜发展多种形式的适度规模经营，继续推进集体林权制度改革，规范海域使用权流转。搭建运作规范、群众信任的土地流转平台，促进土地承包经营权有序流转。

（七）推进新型城镇化建设。深入实施新型城镇化建设行动和城市精细化管理行动，扎实推进和谐城乡建设。

加强园区和小城镇建设。充分发挥规划的引领调控作用，进一步完善城乡规划体系，统筹区域协调发展。中心市区加快“两城两区”开发建设，双岛湾休闲旅游度假城和临港产业服务区在坚持高标准规划的同时，抓紧实施基础设施建设。高区、经区提升发展层次，工业新区加强项目引进。各市区要以强化产业支撑为重点，科学推进城区、新区开发，大力发展县域经济。落实扩权强镇政策，推动1个省级、4个市级试点镇集聚发展。

推动城市精细化管理。理顺城市管理体制，建设城市管理信息平台，在市区70平方公里区域推行网格化的城市管理新模式，实现城市管理的规范化、标准化和信息化。推进市政道路等城市公共设施管理养护体制改革。持续开展停车秩序、机动车尾气、工地扬尘、户外广告等综合整治。建立市区城市建设项目交通压力评估制度，加大投资力度，实施公交优先发展战略，加快建设绿色公交走廊示范项目，进一步改善市民出行环境。强化街道、社区在城市管理中的基础地位，推进城乡和谐社区建设。

（八）强化资源环境保护。实施最严格的节能减排约束机制、最严格的水资源管理制度和最严格的土地矿产资源管理措施，积极转变资源开发利用方式，推进生态文明建设。

大力推进节能减排。综合运用超标准耗能加价、差别电价等调控手段，加强重点用能企业的能源监察审计和重点排污企业的在线监测，强化企业的环境监管。突出抓好工业、建筑、交通、公共机构等领域的节能工作，加快淘汰落后产能，推进电厂脱硫脱氮工程，在生产生活领域推广利用太阳能。完善再生资源回收利用和中水回用网络。加快推进荣成石岛、文登南海、乳山银滩3个污水处理厂建设，推动污水处理厂污泥和垃圾渗滤液处置工程尽快投入运行，积极开展市区生活垃圾分类收集试点，实现垃圾处理的无害化、减量化、资源化。

强化资源保护和集约利用。加快建立健全规划控制机制，加强土地、海域、岸线、沙滩、山体、林地、河流、地热等自然资源保护和统筹利用。完善用海制度，加大海岸带综合管理力度，严厉查处污染海域、破坏沙滩、私搭乱建等违法行为；切实加强围填海项目管理，整治修复重点海域和岸线；规范近岸海域养殖管理，保障群众正常亲海活动。落实耕地保护和节约集约用地制度，加强土地综合整治，实施好城乡建设用地增减挂钩工作，依法清理处置闲置用地。严厉打击非法采砂，关闭市区48家采石场，做好生态修复治理。加强集中式饮用水水源地保护，启动米山水库、崮山水库人工湿地净化工程，抓好母猪河、乳山河流域和市区9条河道的治理。出台管理办法，规范地热资源开发利用秩序。加强城市地下空间规划，提高人防工程综合利用水平。

加强农村环境保护。按照“清洁家园、清洁田园、清洁水源”的要求，切实改善农村生态环境。推进镇级垃圾中转站建设，保证所有镇都建成垃圾中转站，并配齐必要的运输设备，全面实行垃圾“村收集、镇清运、县处理”。强化农村河道的综合整治，推进镇村污水处理设施建设，年底前所有镇驻地的污水全部实现集中处理。加强农村饮用水水源地保护，确保农村饮水安全。进一步加大农村面源污染、养殖污染的治理力度，积极推进秸秆综合利用。

加强生态市建设。注重生态建设与生态产业发展相结合，加快发展循环经济。大力推进造林绿化，集中力量抓好干线公路绿化工程，加强松材线虫、美国白蛾等病虫害防控，新增成片造林8万亩。加快推进荣成大天鹅生态保护区建设。力争通过国家环保模范城市复核，乳山生态市创建工作通过国家验收，高区、经区争创国家生态工业示范园区。

（九）切实保障和改善民生。全力解决好人民群众关心的问题，把发展的目的真正落实到满足人民需要、实现人民利益、提高人民生活水平上。

积极扩大就业。落实鼓励和促进就业创业的各项政策，强化职业技能培训和公共就业服务，促进创业带动就业。完善困难群众就业援助制度，做好大中专毕业生就业工作，全年新增城镇就业3万人，转移农村劳动力8000人，保持就业形势稳定。健全完善企业工资集体协商制度，建立和谐劳动关系。提高企业最低工资标准，强化监察执法服务，保障劳动者的合法权益。

优先发展教育。贯彻落实《国家中长期教育改革和发展规划纲要》，努力办好人民满意教育。强化教师队伍建设，切实提高教育质量。坚持政府主导、社会参与的原则，办好学前教育，大力发展公办幼儿园，推进城镇小区配套幼儿园建设，创建8处市级“农村学前教育示范镇”。推进义务教育高位优质均衡发展，70%的中小学完成标准化学校建设。加强职业教育基础能力建设，做好国家级、省级职业教育改革发展示范学校创建工作，建设5处职业教育示范实训基地。继续加强与山大、哈工大等高校的共建。统一并提高城乡义务教育生均公用经费标准，加大对困难家庭学生资助力度。进一步健全长效机制，切实加强校园安保工作。

提高社会保障水平。继续做好社会保险省级、市级统筹工作，提高新农合补助标准，加快推进各类保险扩面提标，争取在全省率先实现城乡社会保障全覆盖。进一步提高各项社会救助标准，扩大覆盖面，农村低保标准提高到每人每年1650元，城市低保标准提高到每人每月360元，五保集中和分散供养标准分别提高800元和400元，确保应保尽保。对先天性心脏病、先天失明孤残儿童和贫困白内障患者继续安排免费手术。

加快医疗卫生事业发展。深化医药卫生体制改革，加快基本药物制度实施进度，政府办基层医疗卫生机构全部实行基本药物零差率销售。改革公立医院管理体制、运行机制和监管机制，引导社会资本进入医药服务市场，创办面向不同消费群体的医疗机构。完善基层医疗卫生服务体系，进一步改善镇村医疗条件，加强农村医生队伍建设。落实扶持中医药事业发展的意见，健全中医药服务体系。实施好妇幼保健、健康教育等基本公共卫生服务项目，加强传染病的防控。

加大住房保障力度。推动房地产行业健康平稳发展，加快保障性住房建设，提供经济适用住房450套、公共租赁住房3000套，新增廉租住房货币化补贴180户，帮助中低收入家庭解决住房困难。

（十）大力发展各项社会事业。进一步健全公共文化服务体系，繁荣广播电视、新闻出版事业，加强文化市场管理和文物保护，继续开展好幸福威海系列文化活动。做好台湾梅花鹿和长鬃山羊接受工作。积极争创全国文明城市，深入开展群众性精神文明创建活动。不断完善体育设施，筹办好第三届市运会、亚洲霍比帆船锦标赛、国际长距离铁人三项世界杯赛，开展好全民健身活动。完善计划生育利益导向机制，保持低生育水平，提高人口素质。做好史志编修工作，加强档案整理开发利用。抓好双拥共建和优抚安置，加强国防教育、国防动员、民兵预备役和人民防空建设。进一步做好民族、宗教等各项工作。

全面加强社会管理，推进平安威海建设。全力做好新形势下的群众工作，充分发挥信访、人民调解、仲裁、法律服务和法律援助机构的作用，完善利益诉求机制，保障群众合法权益。加强民主法制建设，抓好“六五”普法工作，积极推动法治市区创建活动，发挥好社会组织和社会工作者的积极作用。强化安全生产监管，实施重点领域安全专项整治，坚决遏制重特大事故发生。建立健全应急管理体系，完善应急救援队伍，加强气象预报、防震防灾等监测预警设施建设和应急救援装备配备，提高自然灾害、事故灾难、公共卫生等领域突发事件的应急处置和救援能力。加强社会治安综合治理，打击各类违法犯罪活动，切实维护社会和谐稳定。

四、切实加强政府自身建设

各位代表！新形势、新任务对政府工作提出了新的更高要求，我们将牢记宗旨，恪守职责，以创先争优为动力，以制度建设为保障，努力打造更加敢于担当、更加勇于进取、更加精于服务的现代政府。

（一）实施承诺制度，进一步优化行政服务。坚持全心全意为人民服务，健全政府承诺、部门践诺、群众评诺机制，在政府部门进一步推行服务承诺制度，减少审批事项，提高办事效率，改善服务质量，争创机关服务品牌。继续实行领导干部工作联系点制度，积极主动地为企业和群众搞好服务。加强学习型政府建设，以学习助推解放思想，增强履职践诺、服务发展的能力。

（二）实施公开制度，进一步推进依法行政。大力推行阳光政务，深化政府信息公开，严格执行重大决策听证、重要事项公示、重点情况通报等制度，畅通行风热线、市长公开电话、市长信箱、社情民意调查中心等渠道，提高政府工作透明度和公众参与度。完善政府规范性文件制定工作机制。贯彻实施山东省“五五”依法行政规划，进一步提高执法队伍素质，严格落实行政执法责任制，规范行政处罚自由裁量权，切实做到严格执法、公正执法、文明执法。

（三）实施问责制度，进一步提升行政效能。建立健全抓落实的领导机制和工作机制，加强目标绩效管理考核，切实提高政策执行力和工作落实力。严格执行和不断完善行政问责制度，对关键环节和重点难点问题一抓到底，坚决防止行政不作为、慢作为和乱作为，确保政令畅通、令行禁止，加快打造效能政府。

（四）实施监督制度，进一步规范行政行为。坚决执行市委的决议和决策部署，依法接受人大法律监督和政协民主监督，虚心听取民主党派、工商联、无党派人士和各人民团体的意见。尊重司法监督，认真做好行政复议和行政应诉工作，依法纠正违法或不当行政行为。审计、监察部门要依法实施专门监督。重视网络监督，自觉接受社会公众和新闻媒体的舆论监督。

（五）实施廉政制度，进一步打造廉洁政府。严格执行党风廉政建设责任制，深入开展廉政风险防范管理工作，强化电子监察等科技防腐手段，建立健全反腐倡廉的长效机制。严肃查处教育、医疗、食品药品安全、环境保护、征地拆迁、涉农利益等重点领域的突出问题，坚决纠正损害群众利益的不正之风。清理纠正“小金库”，严格执行“禁酒令”和因公出国管理制度，进一步严肃机关纪律作风，树立为民、务实、清廉、高效的政府形象。

各位代表！站在新的起点，肩负新的任务，我们又踏上了新的征程。让我们高举中国特色社会主义伟大旗帜，紧密团结在以胡锦涛同志为总书记的党中央周围，全面贯彻落实科学发展观，在市委的坚强领导下，团结一心，与时俱进，开拓进取，真抓实干，为建设创新开放宜居幸福的现代化新威海做出新的更大贡献，以优异成绩迎接中国共产党成立90周年！
